# Pulse Wave Analysis in Normal Pregnancy: A Prospective Longitudinal Study

**DOI:** 10.1371/journal.pone.0006134

**Published:** 2009-07-03

**Authors:** Asma Khalil, Eric Jauniaux, Derek Cooper, Kevin Harrington

**Affiliations:** 1 The Homerton University Hospital NHS Trust, Queen Mary and Westfield College, University of London, London, United Kingdom; 2 Academic Department of Obstetrics and Gynaecology, UCL Institute for Women's Health, University College London, London, United Kingdom; 3 King's College Hospital, London, United Kingdom; Institute for Clinical Effectiveness and Health Policy (IECS), Argentina

## Abstract

**Background:**

Outside pregnancy, arterial pulse wave analysis provides valuable information in hypertension and vascular disease. Studies in pregnancy using this technique show that vascular stiffness is raised in women with established pre-eclampsia. We aimed to establish normal ranges for parameters of pulse wave analysis in normal pregnancy and to compare different ethnic groups.

**Methodology/Principal Findings:**

This prospective study was conducted at The Homerton University Hospital, London between January 2006 and March 2007. Using applanation tonometry, the radial artery pulse waveform was recorded and the aortic waveform derived. Augmentation pressure (AP) and Augmentation Index at heart rate 75/min (AIx-75), measures of arterial stiffness, were calculated.

We recruited 665 women with singleton pregnancies. Women who developed pre-eclampsia (n = 24, 3.6%) or gestational hypertension (n = 36, 5.4%) were excluded. We also excluded 47 women with other pregnancy complications or incomplete follow-up, leaving 541 healthy normotensive pregnant women for subsequent analysis. In the overall group of 541 women, there were no significant changes in AP or AIx-75 as pregnancy progressed. In 45 women followed longitudinally, AP and AIx-75 fell significantly from the first to the second trimester, then rose again in the third (*P*<0.001). The two main ethnic groups represented were Caucasian (n = 229) and Afrocaribbean (n = 216). There were no significant differences in AP or AIx-75 in any trimester between these two ethnic groups.

**Conclusions:**

This study is the largest to date of pulse wave analysis in normal pregnancy, the first to report on a subset of women studied longitudinally, and the first to investigate the effect of ethnicity. These data provide the foundation for further investigation into the potential role of this technique in vascular disorders in pregnancy.

## Introduction

Pulse wave analysis is a non-invasive method of assessing arterial stiffness and other central hemodynamics. [1]–[3] It has become a valuable clinical tool outside pregnancy, particularly in the assessment of conditions such as renal disease, diabetes and atherosclerosis, which have cardiovascular effects. [4]–[10] However, experience in pregnant women is still limited. Early work suggests that this technique could prove valuable in the diagnosis and management of pre-eclampsia and fetal growth restriction. [11]–[13] However, until now, normal values for pregnancy have not been established.

Current obstetric practice relies on the measurement of peripheral blood pressure, but *central* pressure may be more valuable in understanding both cardiac and vascular pathophysiology. The use of sphygmomanometry - which provides only the peak pressure of systole and the nadir of diastole - means that much of the information contained in the shape of the arterial waveform is lost.

Two previous studies have investigated arterial pulse wave parameters in normal human pregnancy. [14], [15] Only one of these studies [15] provided data for the first trimester but the earliest examination was performed at 11 weeks of gestation which corresponds to the end of the third month of pregnancy. In the other [14], only 20 women were studied in each of the three gestational age groups, i.e. 17–20 weeks, 25–28 weeks, and 33–36 weeks of gestation. Mean values were calculated for all data in a given gestational age window; no longitudinal data for individual women were given. Neither study provided longitudinal data and neither explored the potential effect of ethnicity on pulse wave analysis parameters.

Recent studies using pulse wave analysis have confirmed reduced arterial compliance, i.e. increased arterial stiffness, in women with clinically established pre-eclampsia. [11], [12], [16] So far, no study has compared PWA parameters earlier in pregnancy in women who later developed pre-eclampsia or gestational hypertension with those who remained normotensive throughout pregnancy. If arterial pulse wave analysis is to be used clinically for the assessment and possibly the early screening of pre-eclampsia, normal values throughout pregnancy must first be established.

The aims of this study were to evaluate the changes in pulse wave analysis parameters in normal pregnancy and to investigate whether these parameters are affected by ethnicity.

## Materials and Methods

### Ethics statement

This study was approved by the Camden & Islington Community Local Research Ethics Committee. Written consent was obtained from each woman after receiving written information about the research project.

**Figure 1:**
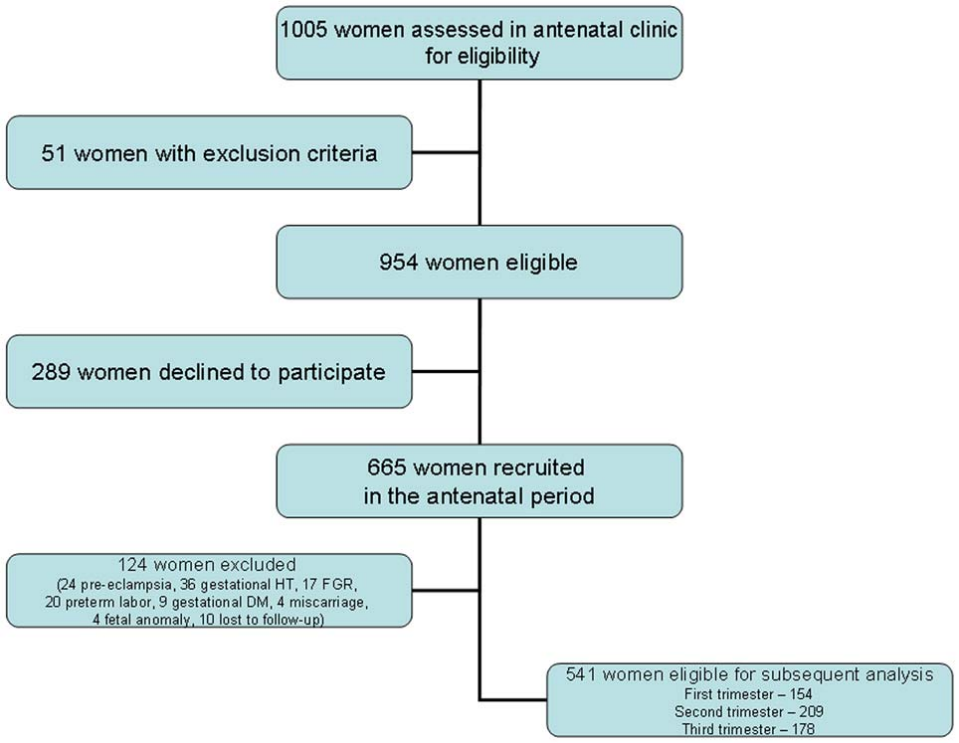
Recruitment and progression of participants through the trial.

This prospective study was carried out at The Homerton University Hospital, London, UK (an associate teaching hospital in an urban setting in London) over a 15 month period between January 2006 and March 2007. During this period, approximately 6,000 deliveries took place. Figure 1 outlines recruitment of women and their flow through the study. During the first six months of the study, women with a singleton pregnancy (n = 1005) attending the antenatal clinic for routine antenatal care were assessed for eligibility. We excluded 51 women with pre-defined exclusion criteria and 289 declined to participate, leaving 665 who were recruited to participate in the study. None of these women had a prior history of cardiovascular disease, chronic hypertension, diabetes, renal disease or immune disorders, and at the time of recruitment none was using medication which could affect blood pressure. Demographic and clinical data including age, body mass index (BMI), parity, blood pressure (BP) and gestational age were recorded. Gestational age was established on the basis of menstrual date and/or ultrasonographic examination prior to 20 weeks of gestation.

All women were followed up until after delivery, and fetal and maternal outcomes were obtained from medical and labour ward records. Pre-eclampsia was defined according to the guidelines of the International Society for the Study of Hypertension in Pregnancy. [17]

All measurements (BP and pulse wave analysis) were performed in the same room at the same temperature (23°C). Participants refrained from caffeine intake on the day of study and rested for at least 10 minutes prior to the measurements. During measurement, the women did not move or speak. Peripheral blood pressure (BP) was measured in duplicate in the brachial artery of the non-dominant arm using a calibrated standard mercury sphygmomanometer. Brachial artery systolic BP was defined using the first Korotkoff sound and diastolic BP using the fifth Korotkoff sound. Mean arterial pressure was calculated by integration of the radial pressure waveform, using the Sphygmocor® system described below. Pulse pressure (PP) was defined as systolic pressure minus diastolic pressure.

**Figure 2:**
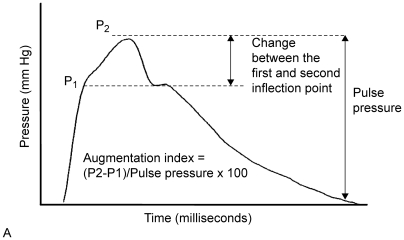
The central (aortic) pulse waveform, represented schematically. A. Typical ascending aortic pulse waveform, showing two systolic peaks (P1 and P2). Augmentation index (AIx) is calculated as the difference between P2 and P1, expressed as percentage of pulse pressure. In hypertensive disorders arterial wall stiffness is increased; the arterial pulse wave travels faster, so the reflected wave reaches the advancing wave in systole, resulting in greater augmentation of the systolic peak. P1 = the first inflection point; P2 = the second inflection point.

Arterial pulse wave analysis was performed as follows: the radial artery was gently compressed with the tip of a tonometer at the site of maximal pulsation. The tonometer contains a micromanometer which provides a very accurate recording of the pressure within the artery (Millar Instruments, Houston, Texas, USA). [2] A generalized transfer function was applied to the radial artery waveform in order to derive the aortic pressure waveform. [18]–[20] From this aortic pressure waveform, the augmentation pressure (AP) and augmentation index (AIx) were calculated. The AP is defined as the height of the late systolic peak above the inflection point on the waveform (Figure 2) and may be positive or negative depending on the relative heights of the two peaks. The AIx is defined as AP expressed as a percentage of the aortic pulse pressure so will be positive or negative depending on the AP. [1], [21] As there is a linear relationship between heart rate and augmentation index, the augmentation index was standardized to a heart rate of 75 bpm (AIx-75). [22] The Sphygmocor® (Atcor Medical, West Ryde, Australia) [1], [21], [23] system was used for analysis of the radial pressure wave contour. All measurements were made by the same investigator (AK). Prior to commencing this study, there was an initial learning period of 25 repeated measurements until satisfactory reproducibility was achieved (<5% variability between duplicate measurements). As a further check, the Sphygmocor® software incorporates a quality control feature which is displayed on the screen.

We calculated that 144 measurements in each trimester would give us the power to be 95% certain that we had estimated the mean AIx75 within each trimester within 2 of the correct value. For the cross-sectional study, only one measurement (the first) from each woman was included for analysis. Baseline characteristics (age, body mass index, parity, ethnicity, smoking status) were compared between the two major ethnic groups in our population, i.e. Caucasian and Afrocaribbean, using the Chi-square test (Fisher's exact test when appropriate) for categorical variables and independent *t*-test for continuous variables. Independent *t*-test was used to compare hemodynamic parameters between these two ethnic groups. For comparison of the different gestational age intervals, we used oneway ANOVA with Tukey HSD post hoc testing to perform the pairwise comparisons. Pearson correlation, *t*-tests and oneway ANOVA were used to analyze the associations between pulse wave analysis parameters (AP and AIx-75) and both baseline characteristics and other hemodynamic parameters (age, BMI, parity, smoking, systolic BP, diastolic BP).

Fetal growth restriction was defined as birth weight less than the 5^th^ centile for gestational age.

Longitudinal data were analyzed using repeated measures ANOVA with the Bonferroni post-hoc test for pairwise comparisons. Binary logistic regression was used to compare Caucasian and Afrocaribbean groups to take account of BMI and parity which were significantly different between the groups. A value of *P*<0.05 was considered to be statistically significant. All *P* values were two-tailed. Data were analyzed using SPSS® (SPSS version 16.0.2, 2008, SPSS Inc., Chicago, IL, USA) and GraphPad Prism 5 (InStata, GraphPad, San Diego, California, USA).

## Results

Of the 665 pregnant women studied, 24 (3.6%) developed pre-eclampsia, 36 (5.4%) non-proteinuric gestational hypertension and 17 (2.6%) fetal growth restriction (Figure 1). We excluded 20 women (3.0%) with spontaneous preterm labor, 9 (1.3%) with gestational diabetes, 4 (0.6%) who had a miscarriage, 4 (0.6%) who had fetal abnormalities who underwent termination of pregnancy and 10 (1.5%) whose outcomes were missing. This left 541 healthy normotensive pregnant women eligible for subsequent analysis. Of these 541 women, 154 were recruited in the first and early second trimester (8^+1^ to 13^+6^ weeks), 209 in the second trimester (14^+0^ to 26^+0^ weeks), and 178 in the late second trimester and third trimester (26^+1^ to 39^+0^ weeks). Of the 154 women recruited in the first trimester, 45 had measurements taken at 12^+0^–12^+6^ weeks, 23^+0^–23^+6^ weeks, and 32^+0^–32^+6^ weeks of gestation; the data from these 45 women were also analyzed longitudinally.

**Figure 3:**
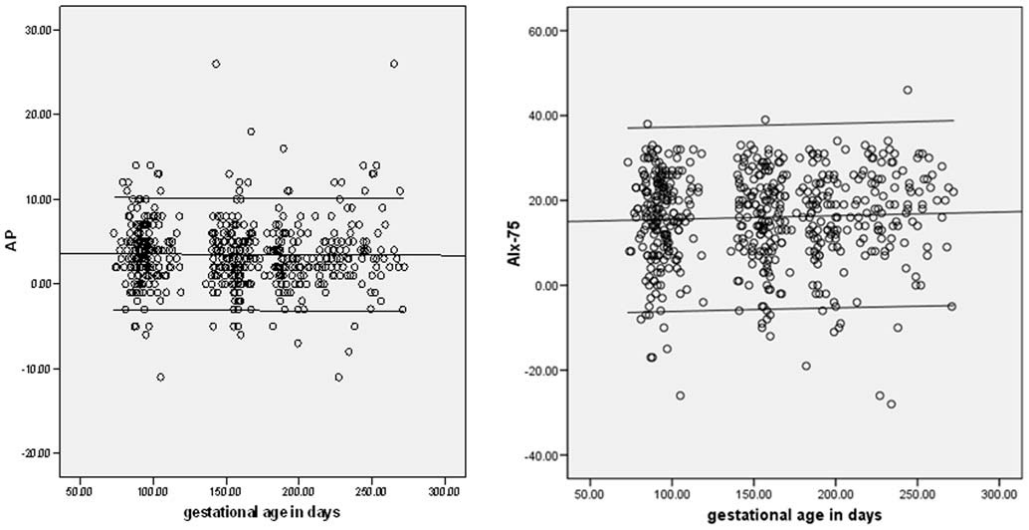
Pulse wave analysis parameters according to gestation. Scatter plots of (a) augmentation pressure (AP), and (b) augmentation index at heart rate of 75/min (AIx-75) according to the gestational age in days (n = 541). The 5^th^ and 95^th^ centiles are shown. Only one measurement from each woman is included.

**Figure 4:**
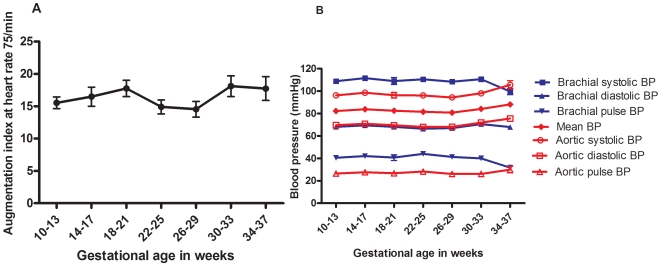
Monthly changes in augmentation index at heart rate of 75/min, central and peripheral blood pressure. Monthly [10^+0^–13^+6^ weeks (n = 145), 14^+0^–17^+6^ weeks (n = 56 ), 18^+0^–21^+6^ weeks (n = 55 ), 22^+0^–25^+6^ weeks (n = 104), 26^+0^–29^+6^ weeks (n = 86), 30^+0^–33^+6^ weeks (n = 57 ) and 34^+0^–37^+6^ weeks of gestation (n = 38)] changes throughout pregnancy in: (a) augmentation index at heart rate 75 beats per minute (AIx-75), and (b) brachial and aortic blood pressures. Values represent mean values and error bars represent standard errors. Only one measurement from each woman is included. BP = blood pressure.

The baseline characteristics of the recruited women are shown in Table 1. There were no significant differences in baseline characteristics among these women recruited in the three trimesters. There was no significant correlation between the estimated means for AP or AIx75 and age, parity, BMI or smoking. We compared the hemodynamic parameters according to trimester. There were no significant changes in AP or AIx-75 as pregnancy progressed. There was no significant change in either brachial systolic or diastolic BP from trimester to trimester, but brachial pulse pressure was significantly lower in the third trimester compared with the second (*P* = 0.004). Heart rate rose significantly from first to second (*P*<0.001), and second to third trimesters (*P*<0.001). The changes in central diastolic blood pressure approached significance (*P* = 0.055) and the post hoc comparison showed a significant difference between the second and third trimesters (*P* = 0.045), rising from a mean (SD) mmHg of 68.79 (8.65) to 71.07 (10.51). The means and standard deviations for AP and AIx-75 in each trimester are given in Table 2, and the changes according to days of gestation are shown in Figure 3 (AP: r = −0.01, *P* = 0.80; AIx-75: r = 0.04, *P* = 0.34). The monthly changes in AIx-75, brachial and central BP are presented in Figure 4.

**Table 1:** Baseline characteristics of the study groups.

	All	Caucasian	Afrocaribbean	*P* value
	n = 541	n = 229	n = 216	
Age (years)	30.38 (6.12)	31.0 (6.2)	30.4 (6.0)	0.30
BMI (kg/m^2^)	26.84 (4.98)	26.4 (4.6)	27.7 (5.2)	0.004
Nulliparity n (%)	242 (44.8)	115 (50.4%)	85 (39.4%)	0.024
Smokers n (%)	78 (14.4)	31.0 (6.2)	30.4 (6.0)	0.30

BMI = body mass index.

Data are expressed as means±SD or as percentages.

*P* values indicate the difference between the two ethnic groups.

**Table 2:** Mean and standard deviation (SD) of augmentation pressure (AP) and augmentation at heart rate 75 beats per minute (AIx-75) in each trimester.

	First trimester	Second trimester	Third trimester	Total
	n = 154	n = 209	n = 178	n = 541
AP mean (SD)	3.89 (3.77)	3.35 (3.95)	3.35 (4.28)	3.50 (4.02)
AIx-75 mean (SD)	15.82 (10.98)	15.88 (10.60)	16.31 (1.59)	16.00 (11.02)

**Figure 5:**
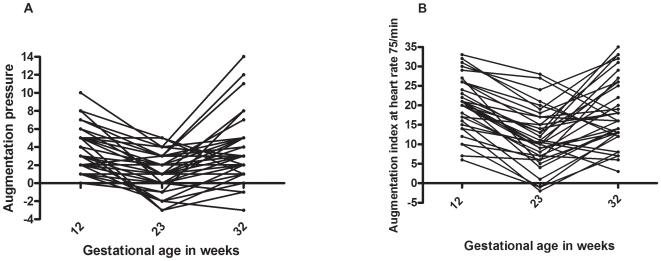
Longitudinal changes in pulse wave analysis parameters. Longitudinal data for the 45 women who had measurements taken at 12^+0^–12^+6^ weeks, 23^+0^–23^+6^ weeks, and 32^+0^–32^+6^ weeks of gestation: (a) augmentation pressure (AP), and (b) augmentation index at heart rate 75 beats per minute (AIx-75).

Figure 5 shows the longitudinal data for the 45 normotensive women who had measurements taken at 12^+0^–12^+6^ weeks, 23^+0^–23^+6^ weeks, and 32^+0^–32^+6^ weeks of gestation. The fall in both AP and AIx-75 in the second trimester and the rise in both parameters in the third trimester were statistically significant (*P*<0.001). There was a non-significant drop in mean blood pressure in the second trimester, and a significant rise again in the third trimester (*P* = 0.015).

We studied 229 Caucasian and 216 Afrocaribbean pregnant normotensive women. Baseline characteristics of these two major ethnic groups were compared including age, BMI, parity, smoking (Table 1). Body mass index was greater (27.70 vs 26.37; *P* = 0.004) and nulliparity less common (39.4% vs 50.4%; *P* = 0.024) in Afrocaribbean compared with Caucasian women. There was no significant difference in any of the hemodynamic parameters between these two ethnic groups (Table 3). After correction for the differences in BMI and nulliparity, the difference in AP and AIx-75 remained non-significant. The numbers of Caucasian and Afrocaribbean women respectively recruited in each trimester were: 11^+0^ to 13^+6^ weeks, 68 and 60; 14^+0^ to 26^+0^ weeks, 87 and 88; 26^+1^ to 33^+0^ weeks, 74 and 68. There were no statistically significant differences in AP or AIx-75 (corrected for BMI and nulliparity) between these two ethnic groups in any trimester.

**Table 3:** Hemodynamic parameters in Caucasian and Afrocaribbean women.

Parameter	Caucasian	Afrocaribbean	P value
	n = 229	n = 216	
Brachial systolic BP (mmHg)	107.4 (12.6)	109.9 (16.7)	0.08
Brachial diastolic BP (mmHg)	67.8 (8.3)	68.4 (9.2)	0.48
Brachial pulse pressure (mmHg)	39.7 (8.8)	41.5 (14.3)	0.10
Mean BP (mmHg)	82.3 (9.9)	83.1 (11.0)	0.39
Heart rate (bpm)	84.8 (14.5)	87.5 (17.5)	0.08
Central systolic BP (mmHg)	96.1 (11.3)	98.1 (15.6)	0.12
Central diastolic BP (mmHg)	69.8 (8.8)	70.2 (9.4)	0.35
Central pulse pressure (mmHg)	27.1 (9.4)	28.2 (10.0)	0.65
AP (mmHg)	3.4 (3.7)	3.5 (4.3)	0.73
AIx-75 (%)	15.6 (11.4)	15.7 (11.2)	0.99

HR = heart rate.

BP = blood pressure.

bpm = beats per minute.

AP = augmentation pressure.

AIx-75 = augmentation index at heart rate 75 beats per minute.

Data are expressed as means±SD or as percentages.

## Discussion

Our study establishes the normal ranges for pulse wave analysis parameters in normal pregnancy. We found no significant differences in these parameters between the two main ethnic groups in our population. Our study confirms that in normal pregnancy, aortic stiffness varies throughout pregnancy, reaching its nadir in the second trimester and rising again in the third. [15] Individual women followed longitudinally throughout pregnancy showed a significant fall in arterial stiffness (AIx-75) in the second trimester, with a significant rise again in the third. These changes were not linked to variations in blood pressure, which in our population did not change significantly through pregnancy, or to changes in heart rate, which rose consistently throughout pregnancy and was controlled for in AIx-75. These results suggest that changes in arterial stiffness may result from changes in the levels of vasoactive substances such as progesterone and relaxin, and volume expansion of pregnancy. When we pooled our data, we found no significant changes in mean AP and AIx-75 from one trimester to the next. However, we observed a trend towards a fall in AIx-75 in the second trimester, and a rise in the third, consistent with the significant trend seen in the sub-group of women followed longitudinally.

Heart rate rose significantly from first to second, and second to third trimesters. AIx has a linear relationship with heart rate, [22] highlighting the importance of controlling augmentation index for pulse rate and thus the need in pregnancy to use AIx-75 rather than AIx.

Each heartbeat generates a pulse wave which travels away from the heart along the arterial tree. This waveform is reflected from bifurcations within the arterial tree and from the junctions of the pre-resistance and resistance vessels. [1], [3], [23], [24]–[26] The reflected wave travels back towards the heart and meets the advancing wave. Thus, the height of the pulse wave at any point in the arterial tree is the net combination of the advancing and reflected waves (Figure 2). Generally, the reflected wave reaches the aorta during diastole, boosting the height of the diastolic portion of the wave. This also helps to maintain coronary artery perfusion.

When arterial wall stiffness is increased, as in hypertensive disorders of pregnancy, the arterial pulse wave travels more rapidly away from the heart and the reflected wave returns more rapidly. [21], [27] As a result, the reflected wave reaches the advancing wave in systole, resulting in significant augmentation of the systolic peak. This can be measured as raised augmentation pressure and augmentation index. The fact that AP and AIx-75 values can be positive or negative contributes to relatively wider standard deviations than might otherwise have been expected.

There is the potential for selection bias in our study. However, we investigated whether the normal ranges of AP and AIx-75 in our study population were influenced by factors that could potentially affect, or have been reported to affect, arterial stiffness; these included ethnicity, age, parity, smoking and BMI (factors which could conceivably have affected the likelihood of having the booking visit in the hospital or community, and thus the chances of being recruited). The normal ranges of either AP or AIx-75 in our study population were not significantly affected by any of these factors. Furthermore, the exclusion of women with any of a long list of high risk factors (which could potentially affect arterial stiffness) should mitigate against selection bias. It is likely, therefore, that the potential for selection bias is low, and the normal ranges we describe should be applicable to low-risk women in other populations.

This study is the largest to date of pulse wave analysis in pregnancy, the first to report on a subset of women studied longitudinally, and the first to investigate the effect of ethnicity. Given that this study excluded women who developed hypertensive disorders of pregnancy, it does not help to explain why Afrocaribbean women are more likely to develop pre-eclampsia. Arterial pulse wave analysis is non-invasive and easy to learn. The equipment is inexpensive and portable and can be easily used in the outpatient setting. This study establishes normal values for pulse wave analysis parameters in all three trimesters and in Caucasian and Afrocaribbean women. These data may be used as the basis for further investigation into the role of pulse wave analysis in the assessment, management and perhaps prediction of pre-eclampsia.
